# Supplementary material for: Pre-Sleep Casein Protein Ingestion Does Not Impact Next-Day Appetite, Energy Intake and Metabolism in Older Individuals
Source: Nutrients. 2019 Dec 28;12(1):90. doi: 10.3390/nu12010090 (PMC7019576; doi:10.3390/nu12010090)
Supplement: Supplementary file 1 [file nutrients-12-00090-s001.pdf]

Supplementary Materials

Table S1. Individual LSEQ scores for each question. Questions are scored from 0-100.

	LSEQ Questions									
	**The way you fell asleep last night**			**Quality of sleep**		**Awakening**		**When waking**	**Now**	**Balance/ Coordination**
**Participant/Condition**	**Slower-Quicker**	**Less-More Sleepy**	**Difficult-Easier**	**More Restless-Calmer**	**More-Less Wakeful Periods**	**Harder-Easier**	**Longer-Quicker**	**Tired-Alert**	**Tired-Alert**	**More-Less Disrupted**
**Subject 1**	**Q1**	**Q2**	**Q3**	**Q4**	**Q5**	**Q6**	**Q7**	**Q8**	**Q9**	**Q10**
**Baseline**	60	60	50	50	50	60	60	60	70	50
**CP**	50	49	50	51	50	40	41	35	36	31
**MD**	79	45	45	44	14	73	74	89	95	51
**WP**	49	48	47	65	77	97	91	80	72	73
**Subject 2**	**Q1**	**Q2**	**Q3**	**Q4**	**Q5**	**Q6**	**Q7**	**Q8**	**Q9**	**Q10**
**Baseline**	62	50	60	62	75	37	37	52	79	54
**CP**	50	51	50	41	40	50	51	62	80	50
**MD**	70	51	66	46	51	59	54	65	74	51
**WP**	73	62	68	70	65	69	52	64	74	63
**Subject 3**	**Q1**	**Q2**	**Q3**	**Q4**	**Q5**	**Q6**	**Q7**	**Q8**	**Q9**	**Q10**
**Baseline**	25	70	25	30	30	50	50	15	20	50
**CP**	26	71	21	17	10	45	43	10	43	16
**MD**	17	49	12	47	7	47	47	3	3	45
**WP**	20	73	8	2	2	51	48	7	6	45
**Subject 4**	**Q1**	**Q2**	**Q3**	**Q4**	**Q5**	**Q6**	**Q7**	**Q8**	**Q9**	**Q10**
**Baseline**	90	93	68	18	11	15	25	38	41	28

**CP**	72	53	61	6	4	57	56	90	93	45
**MD**	81	84	88	13	13	49	49	32	27	49
**WP**	67	71	74	22	19	74	81	67	73	72
**Subject 5**	**Q1**	**Q2**	**Q3**	**Q4**	**Q5**	**Q6**	**Q7**	**Q8**	**Q9**	**Q10**
**Baseline**	44	52	49	36	60	50	50	74	63	49
**CP**	47	49	47	50	49	51	58	34	49	47
**MD**	52	52	52	50	15	50	49	27	31	50
**WP**	51	50	50	60	65	57	58	49	44	47
**Subject 6**	**Q1**	**Q2**	**Q3**	**Q4**	**Q5**	**Q6**	**Q7**	**Q8**	**Q9**	**Q10**
**Baseline**	48	47	48	67	71	50	50	69	72	42
**CP**	55	55	54	70	66	52	52	34	50	50
**MD**	49	32	50	51	51	52	52	67	68	53
**WP**	54	53	53	24	22	52	51	53	19	55
**Subject 7**	**Q1**	**Q2**	**Q3**	**Q4**	**Q5**	**Q6**	**Q7**	**Q8**	**Q9**	**Q10**
**Baseline**	19	19	19	34	26	49	49	40	77	52
**CP**	26	27	27	29	28	50	50	66	68	47
**MD**	27	49	34	36	50	53	54	53	62	50
**WP**	52	73	62	27	23	53	51	78	79	48
**Subject 8**	**Q1**	**Q2**	**Q3**	**Q4**	**Q5**	**Q6**	**Q7**	**Q8**	**Q9**	**Q10**
**Baseline**	52	54	50	50	52	52	54	55	57	54
**CP**	54	55	52	50	52	51	53	67	67	53
**MD**	42	55	45	48	52	55	54	51	59	51
**WP**	50	47	50	51	49	55	48	52	55	54
**Subject 9**	**Q1**	**Q2**	**Q3**	**Q4**	**Q5**	**Q6**	**Q7**	**Q8**	**Q9**	**Q10**
**Baseline**	48	49	48	39	39	51	51	62	83	51
**CP**	44	34	33	34	26	49	49	42	55	40
**MD**	37	50	39	36	56	57	55	69	81	54
**WP**	25	26	24	32	54	50	50	42	43	52

Subject 10	Q1	Q2	Q3	Q4	Q5	Q6	Q7	Q8	Q9	Q10
**Baseline**	48	81	35	29	52	14	13	14	14	36
**CP**	87	85	84	53	78	70	87	70	71	50
**MD**	38	70	66	45	65	68	73	75	75	81
**WP**	40	37	49	58	59	24	54	67	90	86
**Subject 11**	**Q1**	**Q2**	**Q3**	**Q4**	**Q5**	**Q6**	**Q7**	**Q8**	**Q9**	**Q10**
**Baseline**	46	45	45	45	45	45	44	47	49	49
**CP**	38	42	36	55	48	41	37	35	36	33
**MD**	36	62	36	44	44	36	34	43	44	44
**WP**	26	47	28	49	48	20	19	18	16	17
**Subject 12**	**Q1**	**Q2**	**Q3**	**Q4**	**Q5**	**Q6**	**Q7**	**Q8**	**Q9**	**Q10**
**Baseline**	41	50	40	34	39	42	39	39	57	47
**CP**	52	45	59	40	38	40	42	39	58	50
**MD**	52	48	53	58	58	51	50	48	49	50
**WP**	63	55	57	51	49	51	51	59	60	53

Table S2. Habitual dietary protein intakes measured using a 3-day weighed food diary.

Habitual dietary protein intake							
**Participant**	**Weight (kg)**	**Average Breakfast Protein Intake**	**Average Lunch Protein Intake (g)**	**Average Dinner Protein Intake (g)**	**Average Snack Protein Intake (g)**	**Total Average Protein Intake Gross (g)**	**Total Average Protein Intake Relative (g/kg)**
**S01**	91.2	25.2	22.3	34.3	8.3	90.1	1.0
**S02**	70.8	31.7	28.6	29.1	12.3	101.7	1.4
**S03**	85.5	58.9	37.4	49.5	32.3	178.1	2.1
**S04**	66.8	10.1	15.0	18.0	12.6	55.7	0.8
**S05**	57.2	4.9	21.2	19.2	18.5	63.8	1.1

**S06**	58.3	8.2	15.6	18.2	4.3	46.3	0.8
**S07**	67.5	23.5	24.3	29.5	20.3	97.6	1.4
**S08**	83.8	13.0	19.4	25.6	16.8	74.8	0.9
**S09**	87.5	21.7	29.9	32.2	20.1	103.9	1.2
**S10**	67.7	20.6	18.3	21.4	16.4	76.7	1.1
**S11**	71.6	12.3	17.4	19.2	14.4	63.3	0.9
**12**	44.7	23.5	22.2	30.1	19.4	95.2	2.1
**AVG**	71.05	21.12	22.64	27.206	16.31	87.26	1.24
**SD**	13.94	14.34	6.58	9.14	7.02	34.35	0.46
